# Network of small no-take marine reserves reveals greater abundance and body size of fisheries target species

**DOI:** 10.1371/journal.pone.0204970

**Published:** 2019-01-10

**Authors:** Fernanda A. Rolim, Tim Langlois, Pedro F. C. Rodrigues, Todd Bond, Fábio S. Motta, Leonardo M. Neves, Otto B. F. Gadig

**Affiliations:** 1 Instituto de Biociências, Universidade Estadual Paulista (UNESP), Campus de Rio Claro, Rio Claro, São Paulo, Brazil; 2 Laboratório de Pesquisa em Elasmobrânquios, Instituto de Biociências, Universidade Estadual Paulista (UNESP), Campus do Litoral Paulista, São Vicente, São Paulo, Brazil; 3 The UWA Oceans Institute and School of Biological Sciences, The University of Western Australia (UWA), Perth, Western Australia, Australia; 4 Laboratório de Ecologia e Conservação Marinha, Instituto do Mar, Universidade Federal de São Paulo (UNIFESP), Santos, São Paulo, Brazil; 5 Departamento de Ciências do Meio Ambiente, Universidade Federal Rural do Rio de Janeiro (UFRRJ), Campus Três Rios, Rio de Janeiro, Brazil; Aristotle University of Thessaloniki, GREECE

## Abstract

No-take marine reserves (NTRs), i.e. areas with total fishing restrictions, have been established worldwide aiming to promote biodiversity and ecosystem conservation. Brazil has 3.3% of its exclusive economic zone protected by 73 different NTRs, however, most of them currently lack scientific knowledge and understanding of their ecological role, particularly regarding rocky reefs in subtropical regions. In this context, this study aimed to contrast a network of NTRs with comparable fished sites across a coastal biogeographic gradient to investigate the effect of fishing and habitat variability on the abundance and body size of rocky reef fish. We used Baited Remote Underwater stereo-Video (stereo-BRUVs) and Diver Operated stereo-Video (stereo-DOVs) systems to simultaneously sample reef fish and habitat. Model selection and results identified habitat and biogeographic variables, such as distance from shore, as important predictor variables, explaining several aspects of the fish assemblage. The effect of protection was important in determining the abundance and body size of targeted species, in particular for epinephelids and carangids. Conversely, species richness was correlated with habitat complexity but not with protection status. This is the first study using these survey methods in the Southwestern Atlantic, demonstrating how a network of NTRs can provide benchmarks for biodiversity conservation and fisheries management.

## Introduction

No-take marine reserves (NTRs) have been established worldwide as an important management strategy, mostly aiming to protect marine biodiversity from the effects of fishing and other human disturbances [1,2]. It is well documented that these NTRs can provide refuge to marine life, increasing local abundance, species richness, body size and the reproductive capacity of fish [3–6]. Networks of NTRs can be used to investigate effects of fishing across biogeographic gradients, with the aim of estimating benchmarks for conservation and fisheries management. Increased biomass of target species has been recorded inside NTRs, contrasting with open areas where the removal of large carnivores can result in higher abundance of prey species, leading to a trophic reorganization. [7–9].

Extensive research has documented that fish assemblage structure varies with physical, chemical and biological factors across biogeographic and habitat gradients [10–12]. In particular, distance from the coast and topographic complexity have shown increase of species richness, abundance and biomass of reef fish [13–19]. It is therefore important for any investigation of the effects of fishing to control for covariates across NTRs and open areas.

Brazil has 8500 km of coastline and a territorial sea that, together with the Exclusive Economic Zone, encompasses 4 million km^2^. Of this area, 26.4% is currently protected by 177 marine protected areas (MPAs), of which 73 are NTRs, representing 3.3% of the country's marine waters [20]. However, the majority of this protection is in large and remote offshore areas, with only 0.3% of these NTRs occurring in small to medium-sized protected areas (1-100km^2^) in coastal waters [20]. The effectiveness of these remote NTRs in terms of achieving conservation objectives has been questioned due to the difficulties of enforcement and monitoring of offshore waters [21,22]. Despite the relatively small sizes of these coastal networks of NTRs, they have potentially high ecological and social value given the greater human impacts occurring in these coastal waters [13,23,24].

Coastal habitats along the northern coast of Brazil (north of 19°S) are dominated by coral reefs, whereas southern regions (between 19–28°S) are typified by rocky reefs. In general, the Brazilian province shelters a high number of endemic species and biomass of marine organisms [25–28]. In the transition zone between tropical and subtropical-temperate environments (20°S to 23°S), the mosaic of habitat types results in one of the highest species diversity of benthic [29] and reef fish species recorded in Brazil [25,28,30]. These transitional reefs are biologically rich and complex environments, where it is vitally important to establish, enforce and understand the benefits of NTRs. However, the few studies available about the effects of Brazilian NTRs on fish assemblage are concentrated in the northern [31] and southern region [32,33] of the country's coastline, or in offshore islands [34,35], with a lack of studies in the transition zones between tropical and subtropical realms of coastal NTR networks.

Historically, NTRs and reef ecosystems in the Southwestern Atlantic have been assessed using underwater visual census (UVC) (e.g. [31,32,34,35]). Despite the benefits of UVCs, such as being a rapid and effective tool in providing precise data especially about conspicuous and sedentary fish species [36–38], biases involving interobserver variability, underrepresentation of large and mobile species targeted by fisheries, as well as inaccuracy of abundance and size estimates can occur [39–41]. In order to mitigate some of these issues and complement fish assemblage assessments, the use of video-based methods to collect data has been increasingly adopted; aided by rapid advancements in video technology and accessibility to cheaper and higher quality equipment [41,42]. Importantly, methods using such technologies create a permanent record allowing fish identification to be confirmed by experts and revisited when necessary.

Baited Remote Underwater stereo-Video (stereo-BRUV) and Diver Operated stereo-Video (stereo-DOV) are being widely employed to assess diverse aspects of fish assemblages [36,43–46]. Stereo-video techniques provide accurate body size and range measurements of individuals from the three-dimensional calibration of imagery [47]. Stereo-BRUV have been found to sample a wide range of species without precluding estimates of herbivorous species [48] and can be applied across a wide variety of habitats and depths [44]. Also, as a remote sensing technique, it detects large and mobile animals which usually avoid divers and active fishing gears [36,43], but has a range of acknowledged biases and limitations related to the presence of the bait and potential underrepresentation of small-bodied fish species (see Langlois et al. [49] and Goetze et al. [36]). Conversely, the presence of a diver may impact the abundance of fish recorded using stereo-DOVs [36,50], suggesting that the combination of methods is more effective to sample fish assemblages [36,51].

In order to expand knowledge about the ichthyofauna of the Southwestern Atlantic, we applied novel non-destructive methods that complement the traditionally used visual sampling techniques, offering potentially more robust estimates of targeted species among protected and fished areas. The improvement of non-lethal and non-destructive techniques to assess fish assemblage is crucial, especially for sensitive habitats inside protected areas such as reef environments, which shelter a significant amount of endangered and endemic species [32]. Thus, this study is the first assessing fish assemblages using stereo-BRUVs and stereo-DOVs in the Southwestern Atlantic, and aims to contribute to the conservation and fisheries management in the region. Based on this, we aim to investigate the response of the fish assemblage to environmental and habitat variables, as well as the effect of protection among NTRs. We hypothesize that: (1) abundance and body size of targeted fish groups will be greater inside NTRs; whereas (2) non-target fish abundance and species richness will be explained better by habitat and biogeographic variables.

## Material and methods

This study was conducted in accordance with all Brazilian government legislation. This includes Federal Government authorization to observe and assess images within the Tupinambás Ecological Station under the permits #48259–1, and also authorization from the São Paulo State government (Fundação Florestal), by the Comissão Técnico Científica—COTEC, to develop the research project.

### Study site

**Fig 1:**
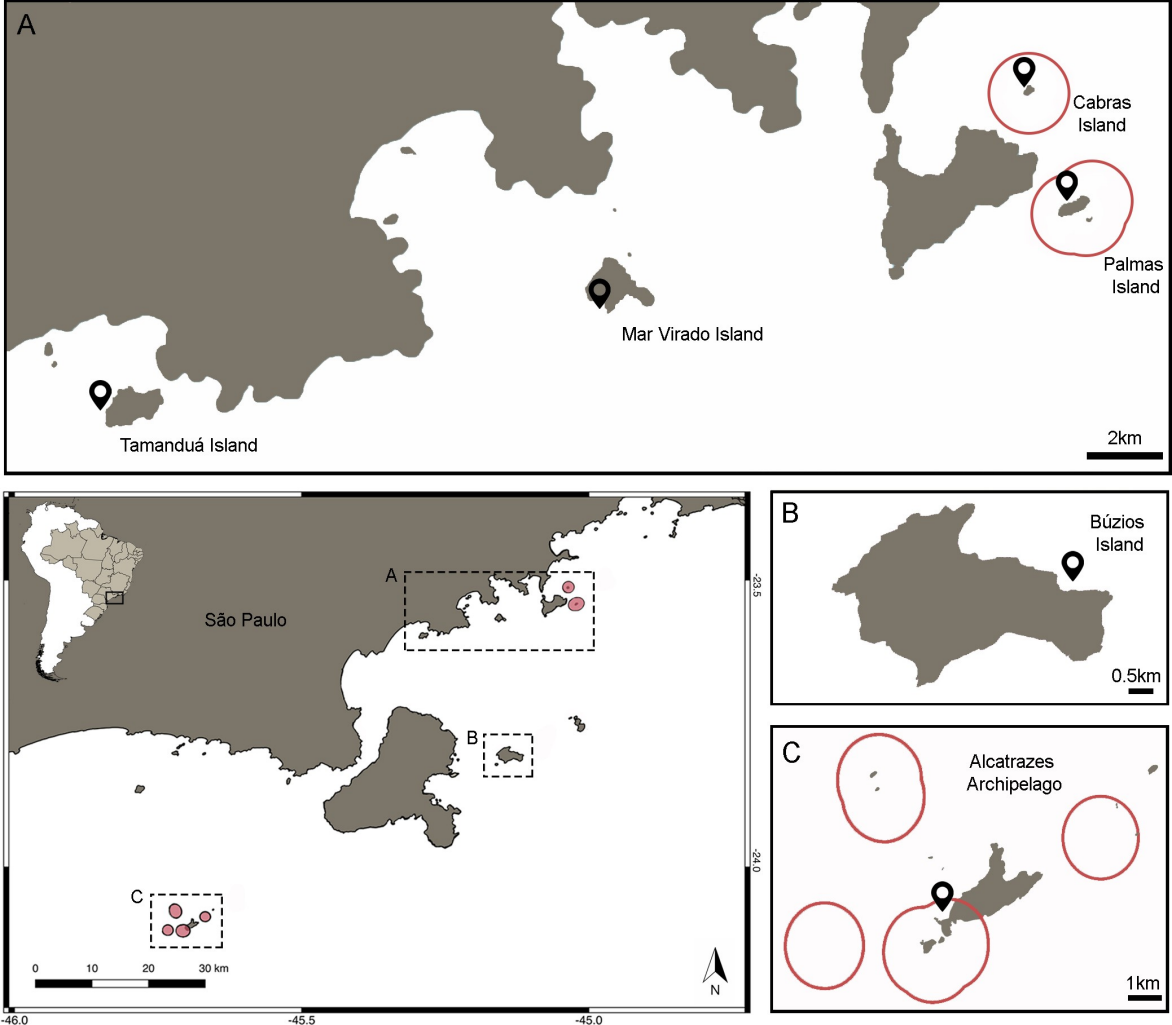
Map of the study area on the southeastern Brazilian coast with the no-take marine reserve Tupinambás Ecological Station in red. The control islands, where fishing activity is permitted (Tamanduá, Mar Virado and Búzios), are also displayed (A, B). No-take areas in detail in the islands of Cabras and Palmas (A) and in Alcatrazes Archipelago (C) with the sample sites represented by the black spots.

The Ecological Station (ESEC) of Tupinambás is a no-take marine reserve (NTR) (corresponding to IUCN Category Ia) located on the northern coast of São Paulo State, Brazil, Southwestern Atlantic. The ESEC was established in 1987 [52] and is divided into two sectors. Sector I is in the archipelago of Alcatrazes (24.101° S; 45.692° W), which is located approximately 43 km from of São Sebastião, São Paulo. This sector has six protected localities, each of them with 1km of buffer area. Two sets of two of these sites are close enough to overlap, creating four primary areas of protection (Fig 1). Sector II protects Palmas Island (23.547° S; 45.029° W) including two nearby reefs (Palmas Reef and Forno Reef) and Cabras Island (23.517° S; 45.041° W), located 5.7 km and 3.6 km respectively from the coast of Ubatuba, São Paulo.

The open-fishing areas used to test the effects of protection on fish assemblage included Búzios (23.804° S; 45.139° W), Mar Virado (23.567° S; 45.156° W) and Tamanduá (23.597° S; 45.289° W) islands. These islands are part of a multiple use MPA established in 2008 (Environmental Protection Area—corresponding to IUCN category V). They are located 34 km, 2 km, and 0.5 km respectively from the mainland. Small scale fishing, such as angling, spearfishing, longlines, fixed traps and gillnetting, is permitted around Mar Virado and Tamanduá islands, but no industrial fishing that uses pair trawling, driftnet vessels above 20 gross tonnage (GT) or trawling vessels up to 10 GT is allowed. However, only pair trawlers are excluded from fishing in Búzios Island.

### Sampling

Samples were collected using stereo-DOVs and stereo-BRUVs. Both equipment types are comprised of a metal base bar with two underwater SeaGIS housing (www.seagis.com.au), each with a digital video camera inside. Housings are positioned approximately 700 mm apart, each inwardly converged at 8 degrees. Stereo-BRUVs were deployed from a boat connected by a rope with a surface float, and left on the seafloor for 90 minutes to record fishes and habitat characteristics. The camera base bar was enclosed within a stainless steel frame, and a bait cage with 800 g of mashed sardine (*Sardinella brasiliensis*) was positioned at the end of a bait arm approximately 1.5 m away from the cameras. Oily bait provide greater sampling efficiency [53,54] due to the odor plume dispersion. Stereo-DOVs used the same camera base bar setup, with the addition of a handle, allowing divers to swim along a transect. These standard survey methods have been developed and used by many authors worldwide [43,44,55].

Stereo-BRUV and stereo-DOV sampling was undertaken bimonthly at each island for a year (2016—March, May, July, October, November; and 2017—January). Each expedition was approximately 4–6 days long, covering all six islands. Due to the small size of islands and to maintain independence among samples (minimum distance between replicates was at least 250 m [48]), only two stereo-BRUV samples were collected on the leeward side of the islands, totaling 12 stereo-BRUVs at each island after six expeditions. Each stereo-BRUV was deployed at the interface of the rocky reef with the sandy bottom. Water depth ranged from 2–17 m depending on the location of the rock-sand interface at each island and the average water depth sampled was 8.3±3.6 m.

Stereo-DOV transects were 25 m long and 5 m wide, and swum at the interface of the rocky shore with the sandy bottom, as well as at the shallow zone above the reef. Due to the small size of the islands, sampling was restricted to three transects at the rock-sand interface and three in the shallow zone on each island at each expedition, totaling 36 transects per island at the end of six expeditions. Stereo-DOV transects were surveyed twice. During the first survey, the observer filmed conspicuous species in the water column; during the second survey, the observer focused on the substrate to detect cryptic species (families Blenniidae, Gobiidae, Labrisomidae and Chaenopsidae). The sampling unit therefore included the number and size of both conspicuous and cryptic fish species per transect. This protocol is comparable to that used for underwater visual census in the region to ensure that the species that are more likely to avoid divers are recorded first, whilst small cryptic species are also sampled [14,18,56]. In stereo-DOV samplings, the interface zone presented an average depth of 8.9±3.8 m and the shallow zone 4.2±1.9 m.

### Video analysis

#### Fish assemblage

Stereo-video systems were calibrated using the CAL software and video analysis was carried out in the EventMeasure software (www.seagis.com.au). The description of the design and calibration of stereo-videos can be found in Harvey and Shortis [47,57]. Fish were identified to the finest taxonomic level possible, counted and measured if they were within 7m of the stereo-BRUVs and 5m for stereo-DOVs.

The relative abundance of each species filmed on stereo-BRUVs was recorded as MaxN, defined as the maximum number of individuals of the same species recorded in a single frame from the left camera. This is a conservative approach in order to avoid counting and measuring the same individual more than once. The fork length of individual fish contributing to a species’ MaxN was measured when the fish was straight and no more than 45 degrees perpendicular to the cameras. In the stereo-DOV, all fish filmed on the left camera were counted and measured using the same rules. These data are stored on GlobalArchive [58] (globalarchive.org), under the project "Effectiveness of Marine Protected Areas, Brazil", and also available in the supporting information files.

Biomass was calculated for all species using measured fish lengths and length-weight relationship referenced in the FishBase database [59]. If equations for fork length of a species were not available, length-length conversions were used if available. Biomass of species without length-weight information was calculated using equations from a similar species from the same family.

Fish species were classified by broad functional groups based on diet, using information available in the literature [60,61] and FishBase [59]. Groups included: carnivores, piscivores, planktivores, roving herbivores, territorial herbivores, omnivores, sessile invertebrate feeders, mobile invertebrate feeders. Piscivores were pooled with carnivores because there was not enough individuals for statistical analysis. Species were categorized in target and non-target for fisheries in the region according to the literature [18,62–64]. Four families (Epinephelidae, Kyphosidae, Scaridae and Carangidae) identified as abundant or frequent and also targeted by fisheries were selected for analysis.

#### Habitat characteristics

Habitat classification and complexity (mean relief) were analysed using a single high definition image of each stereo-BRUV deployment and three single frames of each stereo-DOV transect separated by approximately 8 m. This method is shown to be effective to determine reefs structural complexity [65–67]. Images were analyzed in TransectMeasure software (www.seagis.com.au) using a standardised broad habitat classification scheme based on CATAMI [68] to classify benthic composition and based on Wilson et al. [69] to classify relief characteristics (Table 1). Each image was divided into a 5 x 4 grid and the dominant habitat type of each square was recorded. The proportion of the total number of grid squares that fell on each category was used to estimate percent cover by sample. For stereo-BRUVs, this estimate was based on a single frame per deployment; and for stereo-DOVs it was based on the average of the three replicates per transect. An additional category, ‘reef’, was formed at the end of the image analysis by pooling macroalgae, stony coral, rock and zoanthids, and is based on the similar broad structure these environments present.

**Table 1:** Habitat classification based on broad CATAMI Classification scheme [68] and on Wilson et al. [69], used in Baited Remote Underwater stereo-Videos and Diver Operated stereo-Videos images.

Criteria	Description				
**Relief**	0—Flat substrate, sandy, rubble with few features. ~0 substrate slope				
	1—Some relief features amongst mostly flat substrate/sand/rubble. <45 degree substrate slope				
	2—Mostly relief features amongst some flat substrate or rubble. ~45 substrate slope				
	3—Good relief structure with some overhangs. >45 substrate slope				
	4—High structural complexity, fissures and caves. Vertical wall. ~90 substrate slope				
	5—Exceptional structural complexity, numerous large holes and caves. Vertical wall. ~90 substrate slope				
	Unknown				
**Field of view**	Facing up	Limited			
	Facing down	Open			
**Broad/Benthos**	Ascidians	Consolidated	Open water	Stony corals	Unknown
	Bryozoa	Macroalgae	Sponges	Unconsolidated	Zoanthids

#### Environmental variables

Environmental variables were recorded at each sampling event. Temperature and salinity were measured using a Castaway CTD (Conductivity, Temperature and Depth) and an average temperature and salinity value was calculated from values recorded at the BRUV or dive depth, and 1 m above and below this. Visibility was estimated using a Secchi disk.

### Data analysis

The influence of habitat characteristics and environmental variables on fish assemblage richness, abundance and biomass was investigated using Generalized Additive Mixed Models (GAMM) [70,71] and a full-subsets multiple regression approach based on the function described by Fisher et al. [72]. GAMMs use smoothing splines to estimate non-parametric additive functions, allowing for overdispersion and correlation in the data [70], which may arise in studies like this.

Models were fitted to untransformed overall abundance, richness and biomass data, as well as to abundance by functional group and by families. Models for biomass by functional group and by family were also determined, however, as the same trends were found, we decided to report results on abundance only. A prior selection of the predictor variables was made based on their coverage and on the high collinearity between them (Pearson correlation coefficient r > 0.8). As a result, Reef, Rock and Mean relief remained as continuous variables for the analysis. Null variables of the random model included Month, Method, Depth and Visibility, and fixed factors included Distance to shore (two levels: inshore and offshore) and Protection (two levels: no-take and open). Continuous predictor variables were square root transformed to reduce dispersion of data.

Model selection for each response variable was based on the second-order variant of Akaike’s Information Criterion suited for small samples (AIC_*C*_) [73] and on AIC_*c*_ weights (ωAIC_*c*_). The best model was the most parsimonious one (with the fewest variables) within two AIC_*c*_ units of the lowest AIC_*c*_ value (ΔAIC_*c*_<2) [74]. Because the effect of protection status, and any interactions, were relevant to the primary hypothesis of this study, models that were within two AICc units of the model with the lowest AIC_*c*_ and included protection status, were therefore preferentially investigated (‘hypothesis model’). Selected models had their shape and effective degrees of freedom (EDF) examined to ensure they did not overfit the data.

The distributions of fish lengths for key families were compared inside and outside NTRs using Mann-Whitney *U* test, considering a significant difference as p-values below 0.05. All analyses were performed using R Language for Statistical Computing [75], with the packages gamm4 [76], mgcv [77], MuMIn [78], doParallel [79] and dplyr [80].

## Results

A total of 23,505 individuals were observed belonging to 126 species of 44 families (detailed list in S1 Table, data in S2 Table). Large schools (>100) of sardines (Clupeidae), mullets (*Mugil* spp.), young scads (*Decapterus* spp.), young vermilion snapper (*Rhomboplites aurorubens*) and young grunts (Haemulidae) were excluded from statistical analysis in order to reduce dispersion of data and highlight effects. Not considering these schools, the most abundant and frequent families were grunts (Haemulidae), damselfishes (Pomacentridae), jacks (Carangidae) and snappers (Lutjanidae). The most abundant species were tomtate grunt (*Haemulon aurolineatum*) (28.1%), sergeant major (*Abudefduf saxatilis*) (10.1%), Brazilian damsel (*Stegastes fuscus*) (4.7%), gobies (*Coryphopterus* spp.) (2.3%) and squirrelfish (*Holocentrus adscensionis*) (1.3%). And the most frequent species were tomtate grunt (64.2%), Brazilian damsel (54.2%), sergeant major (49.6%), porkfish (*Anisotremus virginicus*) (38.9%) and dusky grouper (*Mycteroperca marginatus*) (35.1%).

Tomtate grunt was listed as a highly common and abundant species in inshore and offshore areas, and in both no-take and fished areas (Table 2). Brazilian damsel and sergeant major were also highly recorded as abundant and frequent, except for the abundance in offshore no-take areas, which was mainly represented by schools of grunts, scads and vermilion snappers (Table 2).

**Table 2:** Top five most abundant and frequent species (% of samples a species was observed) in no-take and open to fisheries areas in inshore and offshore regions.

	No-take				Open			
	Abundance (n)		Frequency (%)		Abundance (n)		Frequency (%)	
**Inshore**	*Haemulon aurolineatum*	873	*Haemulon aurolineatum*	73	*Haemulon* spp.	651	*Abudefduf saxatilis*	48
	*Abudefduf saxatilis*	604	*Mycteroperca marginatus*	62	*Abudefduf saxatilis*	415	*Stegastes fuscus*	46
	*Decapterus* spp.	500	*Stegastes fuscus*	53	*Stegastes fuscus*	335	*Mycteroperca acutirostris*	35
	*Coryphopterus* spp.	289	*Anisotremus virginicus*	50	*Haemulon aurolineatum*	168	*Haemulon aurolineatum*	26
	*Stegastes fuscus*	265	*Abudefduf saxatilis*	43	*Caranx latus*	96	*Anisotremus virginicus*	22
**Offshore**	*Haemulon aurolineatum*	4336	*Haemulon aurolineatum*	90	*Haemulon aurolineatum*	1231	*Haemulon aurolineatum*	50
	*Decapterus punctatus*	2304	*Pomacanthus paru*	75	*Abudefduf saxatilis*	939	*Abudefduf saxatilis*	56
	*Rhomboplites aurorubens*	1807	*Holocentrus adscensionis*	71	*Haemulon* spp.	500	*Halichoeres poeyi*	33
	*Haemulon* spp.	630	*Kyphosus* spp.	63	*Stegastes fuscus*	285	*Stegastes fuscus*	53
	*Decapterus* spp.	504	*Stegastes fuscus*	60	*Mugil* spp.	264	*Chaetodon striatus*	32

Nineteen species recorded are endemic to the Brazilian Province [28,81–83] and fourteen species are considered threatened (vulnerable/endangered) or near threatened, by the International Union for Conservation of Nature (IUCN) Red List [84] and the Brazilian legislation [85] (detailed list in S1 Table).

**Fig 2:**
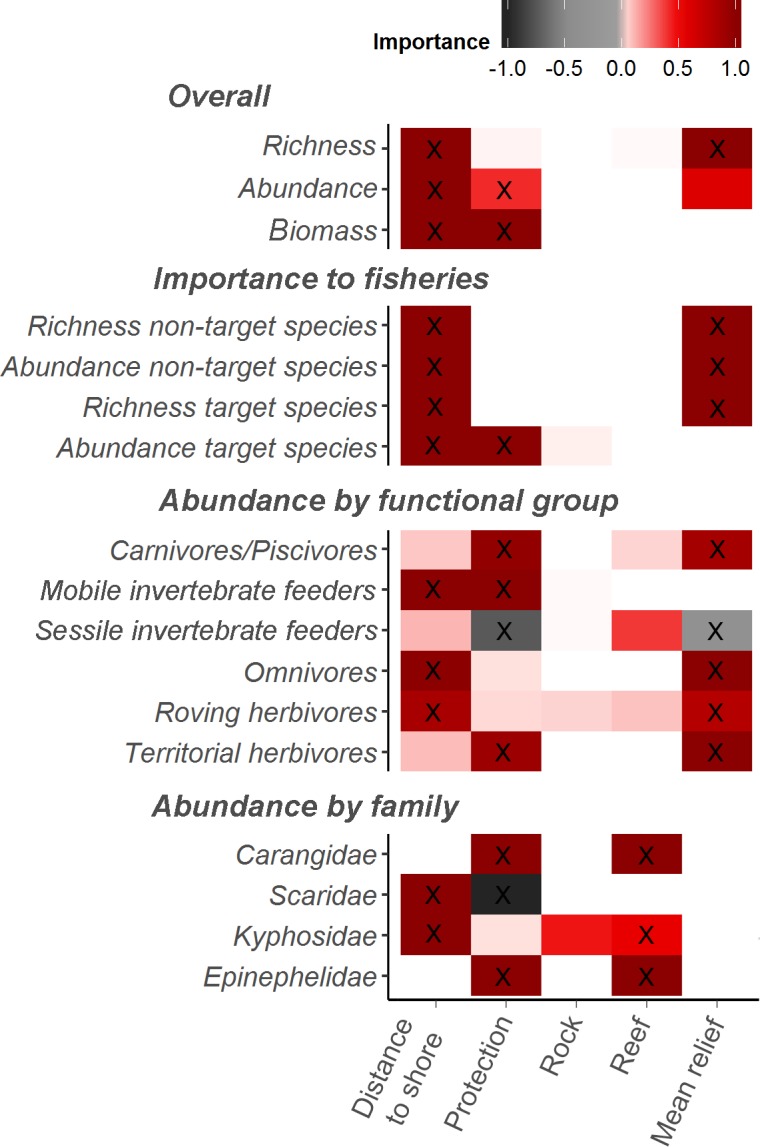
Variable importance scores from full-subset generalised additive mixed models analysis, with >10% variance explained shown. X = Predictor variables within the most parsimonious model for each response variable (see Table 1).

**Fig 3:**
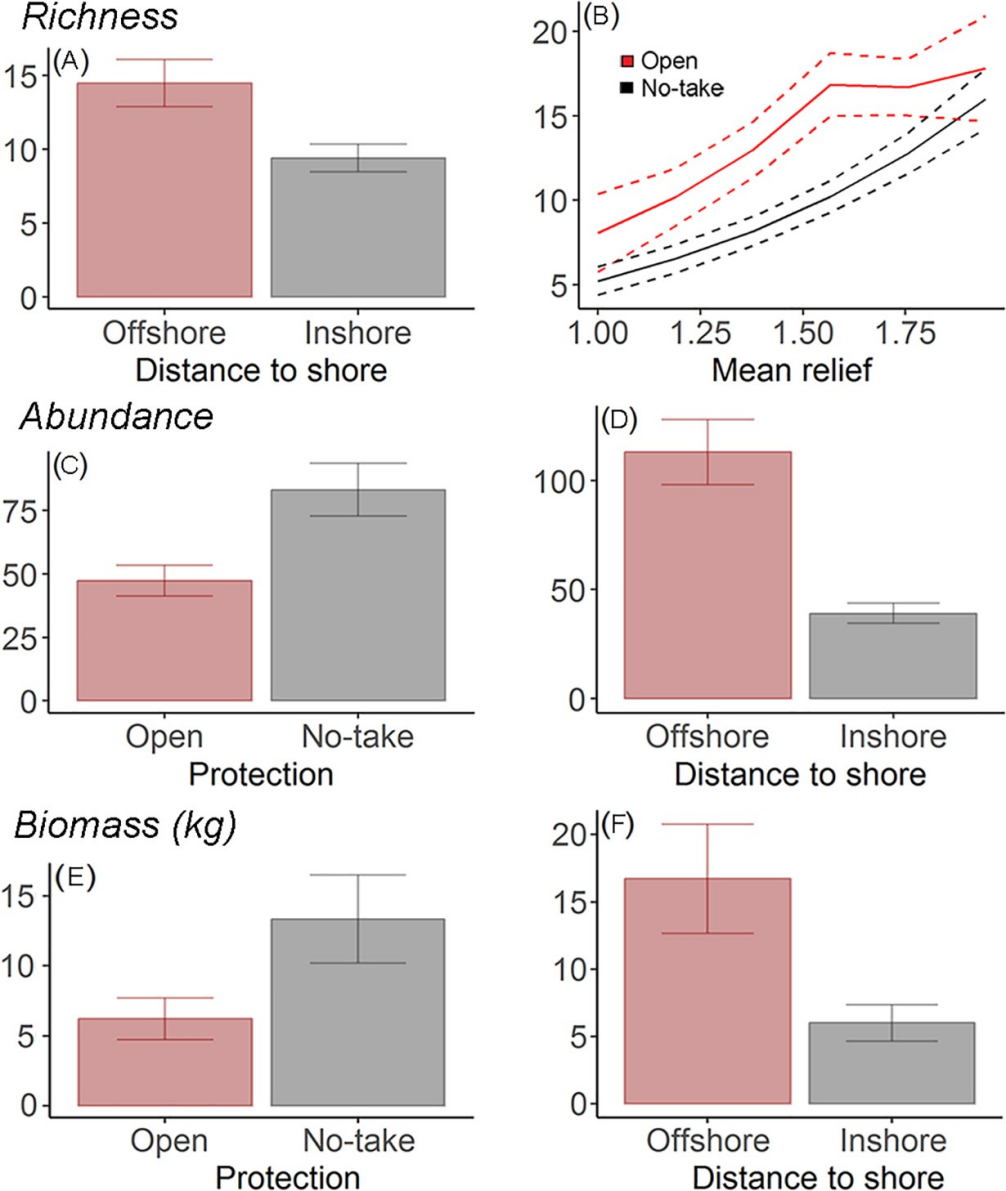
Plots of the most parsimonious models, with >10% variance explained shown. (A,B) species richness, (C,D) total abundance and (E,F) total biomass. The dotted line represents 95% confidence interval.

The most parsimonious model for total richness included distance to shore and mean relief, whereas for both total abundance and biomass the selected models included protection status and distance to shore (Table 3, Figs 2 and 3). The model for overall abundance was selected based on the primary hypothesis of interest, and was within 2AIC of the top model, but it is interesting to note that mean relief was highly important (Fig 2) and present in the most parsimonious model.

**Table 3:** Top generalised additive mixed models (GAMMs) to predict different aspects of fish assemblage.

Dependent variables	Best models	ΔAIC_*c*_	ΔBIC	ωAIC_*c*_	ωBIC	R^2^	EDF
***Overall***							
Richness	Distance to shore + Mean relief.by.Distance to shore	0.00	0.00	1.00	0.89	0.57	18.75
Abundance	Mean relief + Distance to shore	0.49	0.00	0.32	0.68	0.30	8.53
	Protection + Distance to shore	0.00	1.57	0.41	0.31	0.34	8.76
	Distance to shore + Mean relief.by.Distance to shore	0.89	7.52	0.26	0.02	0.30	10.33
Biomass	Protection + Distance to shore	0.00	0.00	1.00	1.00	0.27	16.50
***Importance to fisheries***							
Non-target species richness	Distance to shore + Mean relief.by.Distance to shore	0.00	0.00	1.00	1.00	0.47	14.53
Non-target species abundance	Distance to shore + Mean relief.by.Distance to shore	0.00	4.83	0.90	0.08	0.39	8.54
Target species richness	Mean relief + Distance to shore	0.00	0.00	0.61	0.89	0.56	18.42
Target species abundance	Protection + Distance to shore	0.00	0.00	1.00	1.00	0.25	13.55
***Abundance by functional group***							
Carnivores/Piscivores	Protection + Mean relief.by.Protection	0.00	6.50	0.86	0.02	0.30	17.28
Mobile invertebrate feeders	Protection + Distance to shore	0.00	0.00	1.00	1.00	0.23	10.21
Sessile invertebrate feeders	Protection	1.43	0.00	0.15	0.40	0.13	7.31
	Protection + Reef.by.Protection	0.17	4.73	0.28	0.04	0.16	9.16
	Protection + Mean relief.by.Protection	0.00	12.54	0.31	0.00	0.11	10.92
Omnivores	Mean relief + Distance to shore	0.00	0.00	0.90	0.96	0.31	13.38
Planktivores	Protection + Mean relief.by.Protection	0.00	3.54	0.92	0.14	0.03	8.81
Roving herbivores	Mean relief + Distance to shore	0.00	0.00	0.44	0.48	0.17	6.95
	Distance to shore + Mean relief.by.Distance to shore	0.47	17.10	0.35	0.00	0.18	9.22
Territorial herbivores	Protection + Mean relief.by.Protection	0.00	0.00	0.89	0.93	0.40	14.66
***Abundance by family***							
Epinephelidae	Protection + Reef.by.Protection	0.00	0.00	1.00	1.00	0.27	19.49
Kyphosidae	Distance to shore + Reef.by.Distance to shore	0.00	0.00	0.53	0.42	0.15	17.92
	Distance to shore + Rock.by.Distance to shore	0.30	0.58	0.46	0.31	0.16	17.94
Scaridae	Protection + Distance to shore	0.00	0.00	1.00	1.00	0.10	15.14
Carangidae	Protection + Reef.by.Protection	0.00	0.00	1.00	1.00	0.22	20.22

ΔAICc = Difference between lowest reported corrected Akaike Information Criterion; ΔBIC = Bayesian Information Criterion; ωAICc = AICc weights; ωBIC = BIC weights; R2 = variance explained; EDF = effective degrees of freedom. Model selection was based on the most parsimonious model within two units of the lowest AICc which has the fewest variables.

**Fig 4:**
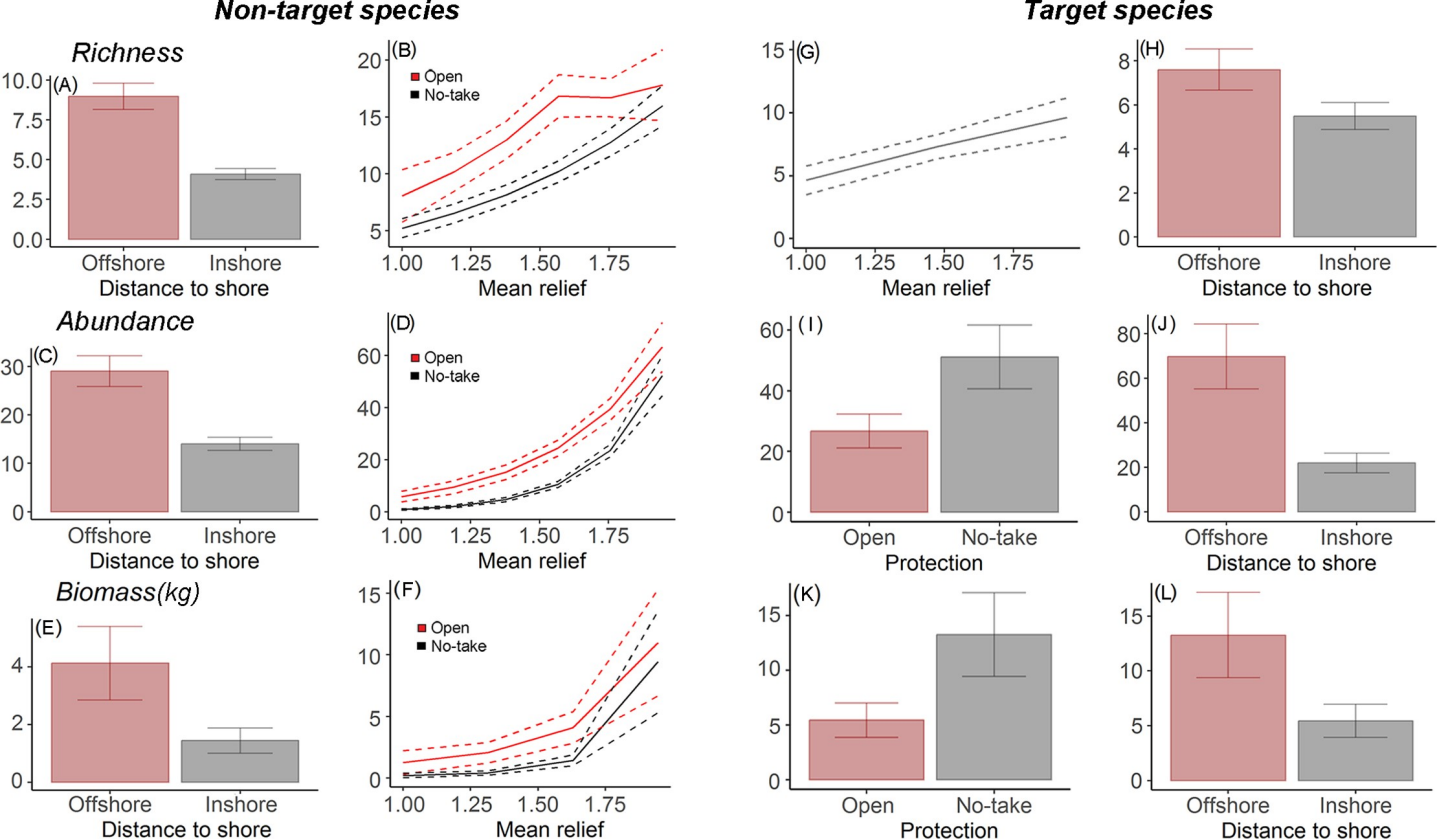
Plots of the most parsimonious models for target and non-target fish assemblage overall characteristics, with >10% variance explained shown. On-target species (A,B) richness, (C,D) abundance, (E,F) biomass. And for target species (G,H) richness, (I,J) abundance, (K,L) biomass. The dotted line represents 95% confidence interval.

**Fig 5:**
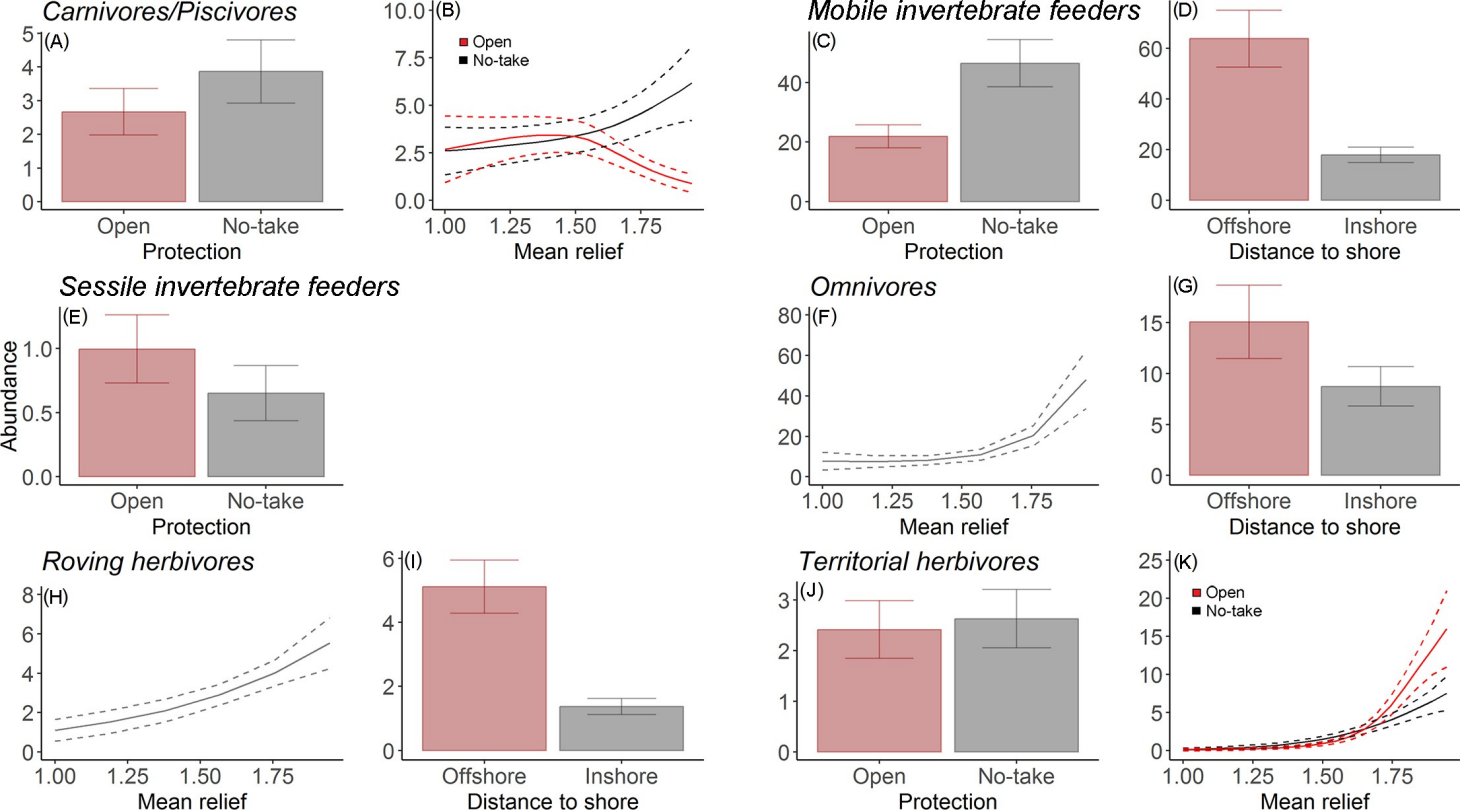
Plots of the most parsimonious model for abundance by functional group, with >10% variance explained shown. (A,B) Carnivores/piscivores, (C,D) Mobile invertebrate feeders, (E) Sessile invertebrate feeders, (F,G) Omnivores, (H,I) Roving herbivores, (J,K) Territorial herbivores. The dotted line represents 95% confidence interval.

Higher richness of target and non-target species and greater abundance of non-target species were best predicted by increased distance to shore and mean relief, whilst the most parsimonious models for the abundance of target species indicated they were likely to increase with protection and distance to shore (Fig 4). Concerning abundance by functional groups, the most parsimonious models for carnivores/piscivores, planktivores and territorial herbivores all included a positive relationship with protection and mean relief. However, as the variance explained by the model for planktivores was very low (R^2^<10) (Table 2), it was not represented graphically in Figs 2 and 5. Contrary to the trend found for the other functional groups, the abundance of sessile invertebrate feeders was found to be negatively correlated with protection status and mean relief. For mobile invertebrate feeders, the abundance is likely to increase with protection and distance to shore. The number of herbivores and omnivores was higher in areas further from shore and also on structurally complex reefs (Fig 5). All data used to fit the models is available on S3 Table.

**Fig 6:**
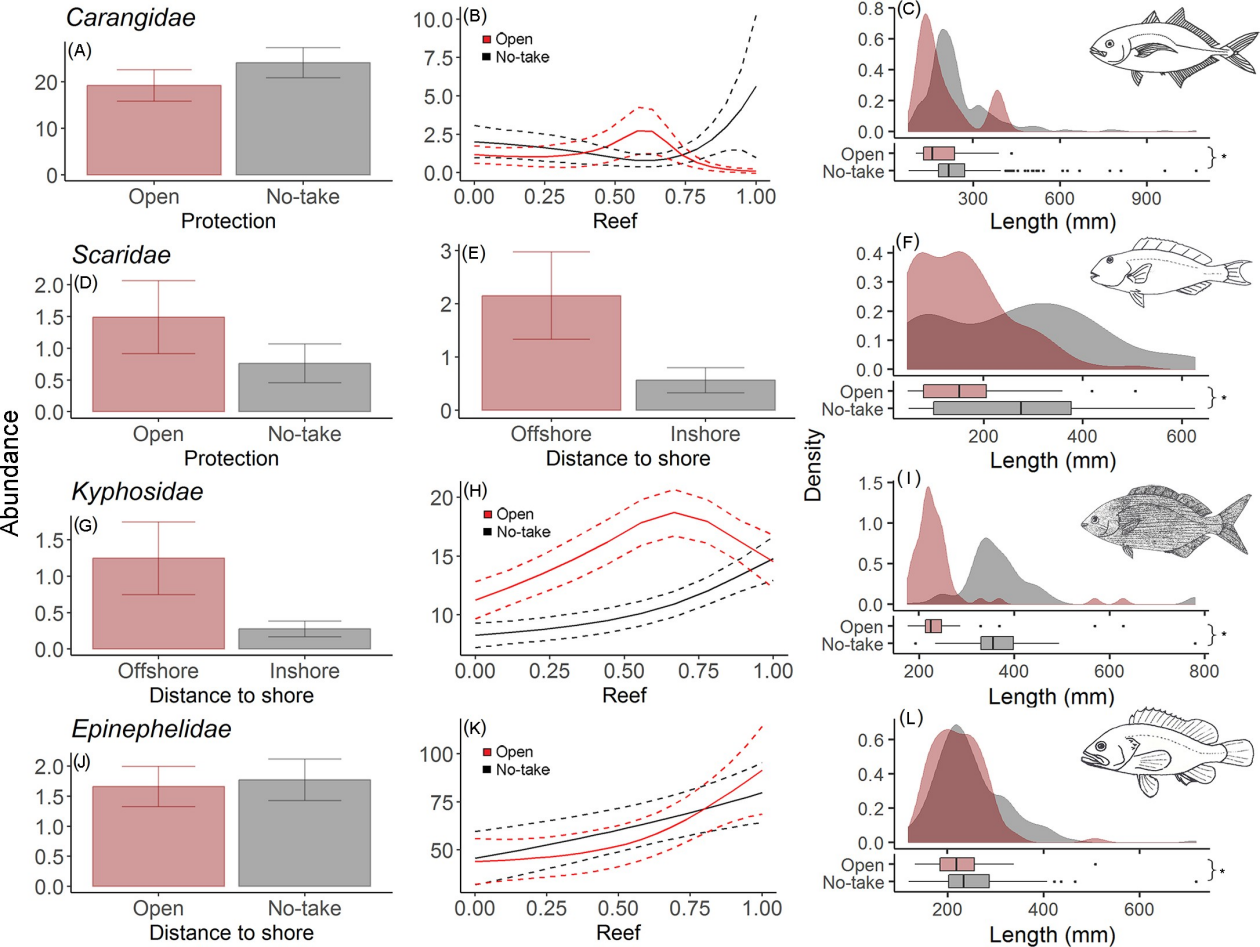
Plots of the most parsimonious models for abundance, Kernel density plots and boxplots for fork length (mm) for important fishing target families. (A,B,C) Carangidae, (D,E,F) Scaridae, (G,H,I) Kyphosidae and (J,K,L) Epinephelidae. The dotted line represents 95% confidence interval. * Significant difference. Fish drawings were based on Carvalho-Filho [61].

Targeted families Carangidae and Epinephelidae increased with protection and presence of reef, whilst kyphosids were found in greater abundance in areas with more reef and greater distance from shore. Scarid abundance showed a negative correlation with protection and a positive correlation with distance to shore (Fig 6). In terms of body size of these families, the largest individuals were found inside the NTRs, with significant differences (Carangidae: *U* = 38283, p-value<0.001; Scaridae: *U* = 4462.0, p-value<0.001; Kyphosidae: *U* = 6450.5, p-value<0.001; Epinephelidae: *U* = 9341.5, p-value = 0.013) (Fig 6C, 6F and 6I).

## Discussion

This study is the first to generate fisheries independent data using non-destructive stereo-video methods in the Southwestern Atlantic. Besides, the approach adopted here made it possible to distinguish the effect of fishing from habitat variables on different components of the fish assemblage, demonstrating how NTRs can be used as benchmarks to contribute to resource management and marine conservation.

### The role of no-take marine reserves

Broadly, total abundance and biomass were greater inside no-take areas, a pattern also registered in previous studies [7,86,87]. The assessment of biomass in the marine environment is important and can reveal the health status of an environment especially because it can be used to represent the energy flux, as well as the potential of the ecosystem to provide goods and services [88,89]. Based on this, the results indicate that the NTR in question is protecting natural processes and resources, which are being effectively converted into biomass. Conversely, the opposite was found in areas open to fishing, presenting a decreased ecosystem functioning driven by the selective removal of large individuals [90,91]. Higher overall abundance and biomass within NTRs indicates the significant removal of fish by fisheries in the open access areas in the region.

Distance from the coast was an important factor, explaining the higher richness, abundance and biomass recorded in islands further from the coast. This factor has been demonstrated to influence fish assemblages structure in several coral and rocky reefs around the world [92–95] and also in the Brazilian Province [13,15,25,60]. The first hypothesis we raise to explain the higher richness and abundance in offshore islands may be related to the total area of rocky reefs. In the region, offshore reefs are typically deeper and form a larger continuous extensions when compared to inshore reefs that are often interspersed with sandy beaches, probably leading a smaller surface area available for reef fishes. Surface area of reef has been directly attributed to fish assemblage structure in some studies. For example, Francini-Filho and Moura [31] found a more pronounced increase of overall biomass over time in areas adjacent to coral reefs that reach deeper water. Furthermore, Roberts and Ormond [96] registered higher species richness with depth, and Gibran and Moura [60] also detected this tendency for rocky reefs in the same region of the present study. These findings might be due to higher availability of resources and a possible lower competition in offshore islands, especially for space [97].

The second hypothesis to explain the higher values of ecological metrics is related to the proximity of anthropogenic activities. The close proximity of human populations to a fish assemblage causes negative effects and is demonstrated worldwide [5,90,98–100]. Areas close to the mainland are easier to access and tend to have more fishing activities. Nearshore waters (<50 m water depth) of the São Paulo state coast, are highly explored by both artisanal and industrial fishing fleets, with artisanal, low mobility fleets most dominant in water depth <20 m [23]. Coastal regions with high population densities, such as São Paulo, are more exposed to human activities causing disturbances and changes in coastal dynamics, especially concerning the high input of nutrients and pollution through air deposition, river discharges, urban and industrial wastewater effluents, groundwater and surface runoff [24]. These potentially harmful components cause environmental stress and may damage coastal biota directly or indirectly [101]. In addition, areas near the coast also face greater exposure to major developments, such as harbors and marinas, which can also significantly change the coastal landscape, causing degradation of habitats and consequently affecting fish assemblage. Further studies in the region are needed to test these hypotheses in order to determine whether or how much of this pattern is explained by biogeography or anthropogenic activities.

### Target and non-target species

Higher abundance of target species was observed within NTRs, but protection status did not correlate with any differences in the abundance of non-target species. Indeed, studies have shown increased abundance of highly targeted fishes inside no-take NTRs, with lower influence on non-target [5,7,35,102,103], reinforcing evidence of the direct effects of fishing. Abundance of target species also increased with greater distance from the shore, which can be related to the increased fisheries activity close to the shore as described above [23].

Conversely, species richness and richness of target and non-target fish, was not related to protection status, being mostly explained by relief. Higher species diversity in more complex environments has been described in the literature [5,14,19], and is likely related to increased availability of food, decreased competition, and lower probability of predator-prey encounters [97,104,105]. Structurally complex environments have higher availability and diversity of niches, accommodating a higher number of species in a small area.

### Fish functional groups

Although functional groups responded differently to fishing pressure, we found evidence that protection status affected the trophic structure of the fish assemblage, since carnivores/piscivores, mobile invertebrate feeders, and territorial herbivores were more abundant within the NTR, whilst sessile invertebrate feeders were less abundant. However, protection was not relevant for omnivores and roving herbivores. The abundance of the carnivores/piscivores functional group, which is comprised of species targeted by fisheries in the region [18,62–64], was higher within NTRs. Even though relief was important, it was relevant only when combined with protection. These results suggests that the NTR is facilitating the recovery of high trophic level organisms, which are usually the first group depleted by fisheries [91,106,107].

Some mobile invertebrate feeder species are targeted by fisheries, but are not considered as important to fisheries as carnivores because of their smaller body size, such as haemulids, labrids and small carangids. Nevertheless, protection was still an important factor to predict abundance of this group, suggesting some fishing pressure, albeit less than highly targeted carnivores/piscivores. This might be related to a depletion of top predators, leading to an exploration of lower trophic levels, as already described worldwide [107], including Brazilian coast [108,109]. The other factor strongly affecting abundance of this group is distance to shore, which may be related to the larger rocky reef surface, offering more resources and, consequently, less competition [97]. This is especially important for small and benthic mobile invertebrate feeders of the families Blenniidae and Serranidae, which live closely associated with the substrate [62]. Similar results were found for omnivores, in which higher abundance is more likely to occur in high complex habitats in offshore islands, probably for the same reasons, since this group encompasses blennies, pomacentrids, pomacanthids and species of the order Tetraodontiformes. Although some species within this category are targeted by fisheries (Mugilidae, Sparidae, Ephippidae), protection was not an important factor to determine abundance. This is probably related to the plasticity of the omnivorous diet, which can enable greater resistance to environmental changes (e.g. [110,111]).

As the abundance of sessile invertebrate feeders was very low in samples, the model was not robust. However, lower abundance found within NTRs and in more complex reefs indicated by the model may be related to the elusive behaviour of these species, which usually hide from divers and may not be recorded. Since these species feed on benthic invertebrates generally associated with hard substrate, we would expect a higher abundance in more topographic complex environments. For planktivores, models did not predict the abundance well, most likely because species in this group show highly variable body sizes, occupying very different niches. For example, fish from Echeneidae and Carangidae families are mobile and large-bodied species, occupying the pelagic environment, whilst the species from Pomacentridae and Pempheridae families are small-bodied species that live associated with burrows and crevices on the rocky reef [62]. Therefore, it was not possible to determine a single robust model to explain abundance of this functional group with the predictor variables used.

Abundance of roving herbivores was related to distance from shore and topographic complexity, which is expected considering its diet, algae and detritus, are mostly found in reef environments [112], which are more likely abundant in larger rocky reefs of offshore islands. This is similar with the results for territorial herbivores, in which protection was only important when combined with topographic complexity. This is also likely, since territorial herbivores, such as damselfishes (*Stegastes* spp.), are found in complex regions of the reef protecting colonies of the major components of their diet, primarily fast growing red and green filamentous algae [113,114]. As habitat characteristics were more influential in herbivores abundance than protection status, fisheries effects were not evident for these groups, even though some of them are targeted in the region.

### Targeted fish families

One of the consequences of large removal of individuals by fisheries activities is represented by a rapid decrease in abundance and richness, especially of large bodied target species [91]. Indeed, the effects of fishing on the size of individuals is well described, in which target species reach larger sizes within NTRs [35,99,102,115,116]. The present study corroborates these findings, showing a significantly higher density of larger individuals of target species of the families Epinephelidae, Kyphosidae, Carangidae and Scaridae within protected areas. This also represents an increase in reproduction capacity of these groups in protected areas as larger individuals usually present much higher fecundity [117]. This increases the probability of exporting larvae from NTRs to adjacent areas [31,118] repopulating fished reefs and helping to restock targeted species in fished areas.

Networks of moderate size (10–100 km^2^) NTRs have demonstrated to be more effective in resource management and conservation when compared to smaller protected areas [119]. However, small (1–5 km^2^) and very small (<1 km^2^) areas have been widely implemented and shown to have some advantages, specifically for small bodied and sedentary species with smaller home ranges [120–123]. In particular, individuals of the Epinephelidae family presented a higher abundance with protection and also in complex environments within the very small NTR in question. These species live associated with burrows within rocky reefs [124] and are highly targeted by fisheries, indicating that they may be the group benefiting most from protection, as seen in this study.

The abundance of kyphosids was not related to protection and was more abundant in regions offshore with the greater presence of reef. As this species is considered herbivorous, grazing predominately on macroalgae (*Sargassum* spp.) associated with rocks [125], we expect to record higher numbers at locations with greater food availability, including offshore areas with more rocky reef. However, larger individuals could be targeted by fishers, resulting in their higher abundance recorded within NTRs. This indicates that the NTRs allow the growth of individuals, and therefore provide greater reproductive capacity for the species.

For the Carangidae family, an effect of protection in abundance was evident, suggesting a high removal, especially of large individuals, in areas open to fisheries. Besides, regardless of being a mobile species, they are frequently found associated with hard structures [45] and even following other species [126], and probably for this reason, individuals of this family have shown to benefit from NTRs in reefs [5,127].

Fish of the Scaridae family showed a higher abundance in fished areas, likely due to the absence of top predators (carnivores/piscivores), since species of this family have been registered to be preyed upon by epinephelids, carangids and muraenids [128]. Even though they were more abundant in fished areas, fish size was smaller, representing a fishing pressure in larger sizes, as also described by Floeter et al. [35]. Also, the abundance of these roving herbivores was higher with distance from the coast, what could be related to the availability of food and lower competition in larger and continuous reefs offshore.

## Conclusions

Brazil shelters the second richest reefs in the Atlantic Ocean [28], and also stands out for the proportion of endemic and endangered species concentrated in small areas [27,28,129]. Therefore, it is crucial to generate information about the role NTRs can play in protecting fish assemblages of this region. In addition, a better understanding of patterns in the effects of fishing on a fish assemblage provides robust metrics for conservation and fisheries management, whilst also providing information on focal species and biological variables most relevant to monitor the effectiveness of NTRs to protect fish assemblages.

The present study presents evidence that very small NTRs (<1km^2^) can protect fish assemblages from the direct effects of fishing, increasing abundance and biomass, especially of targeted species, therefore contributing to the management of fisheries resources at a local and regional scale. Some functional groups showed a higher benefit from protection, such as carnivores/piscivores and mobile invertebrate feeders, while others decreased in abundance, such as the Scaridae family. Another outstanding difference is concerning body size, mostly for target species, in which NTRs allow target species to reach larger sizes. The Epinephelidae family showed greater evidence to benefit from these very small NTRs, especially due to its high importance to fisheries and its small home range. However, we recommend that networks of larger NTRs (>10Km^2^) should be established in the region, which would provide a more robust framework for investigating and managing the effects of fishing and informing conservation and fisheries management more broadly.

As a concluding remark, our findings show strong influence of protection, distance from the shore and mean relief on fish assemblage characteristics, in which protected areas further from the human influence and with a higher topographic complexity tend to have greater abundance and biomass of fish. Our results highlight the crucial role these areas play in the conservation and recovery of highly valuable commercial stocks to the fishing activity of the region, displaying the importance of keeping and implementing more NTRs in the region. The use of stereo-videos in this study has shown to be effective and feasible in this region, providing valuable and robust information to aid conservation and fisheries management in Brazil.

## Supporting information

S1 Table List of species found within no-take reserves and fished areas. *Endemic from Brazilian biogeographic province [28, 81–83]; VU_I_ = Vulnerable by International Union for Nature Protection (IUCN) Red List [84]; NT_I_ = Near threatened by IUCN; VU_Br_ = Vulnerable by Brazilian legislation [85]; CR_Br_ = Critically endangered by Brazilian Legislation; Y = Target; N = Non-target; N = Abundance; F% = Frequency.
(DOCX)

S2 Table Fish abundance data. (TXT)

S3 Table Data used to fit the generalised additive mixed models (GAMMs). (TXT)
