# Supplementary material for: Network of small no-take marine reserves reveals greater abundance and body size of fisheries target species
Source: PLoS One. 2019 Jan 10;14(1):e0204970. doi: 10.1371/journal.pone.0204970 (PMC6328244; doi:10.1371/journal.pone.0204970)
Supplement: S1 Table — *Endemic from Brazilian biogeographic province [28, 81–83]; VUI = Vulnerable by International Union for Nature Protection (IUCN) Red List [84]; NTI = Near threatened by IUCN; VUBr = Vulnerable by Brazilian legislation [85]; CRBr = Critically endangered by Brazilian Legislation; Y = Target; N = Non-target; N = Abundance; F% = Frequency. (DOCX) [file pone.0204970.s001.docx]

**Table S1 - List of species found within no-take reserves and fished areas.** *Endemic from Brazilian biogeographic province [28, 81-83]; VU_I_= Vulnerable by International Union for Nature Protection (IUCN) Red List; NT_I_= Near threatened by IUCN [84]; VU_Br_= Vulnerable by Brazilian legislation [85]; CR_Br_= Critically endangered by Brazilian Legislation; Y= Target; N= Non-target; N= Abundance; F%= Frequency.

| **Family** | **Species** | **Functional**  **group** | **Target** | **No-take** | | | | | |  | **Open** | | | | | | | | **TOTAL N** | | **TOTAL F%** |
| --- | --- | --- | --- | --- | --- | --- | --- | --- | --- | --- | --- | --- | --- | --- | --- | --- | --- | --- | --- | --- | --- |
|  |  |  |  | **Inshore** | | **Offshore** | | **Total N** | **Total F%** |  | **Inshore** | | **Offshore** | | **Total N** | | **Total F%** | |  |  |  |
|  |  |  |  | **N** | **F%** | **N** | **F%** |  |  |  | **N** | **F%** | **N** | **F%** |  |  |  |  |  |  |  |
| Dasyatidae | *Hypanus* spp. | Carnivore | Y | 39 | 5.2 | 3 | 4.2 | 42 | 4.9 |  | 5 | 3.1 | 1 | 2.1 | 6 | | 2.8 | | 48 | | 3.82 |
| Dasyatidae | *Hypanus americanus* | Carnivore | Y | 1 | 1.0 | - | 0.0 | 1 | 0.7 |  | - | 0.0 | - | 0.0 | - | | 0.0 | | 1 | | 0.35 |
| Gymnuridae | *Gymnura altavela* (VU_I_;CR_Br_) | Piscivore | Y | 2 | 1.0 | 1 | 2.1 | 3 | 1.4 |  | 1 | 1.0 | - | 0.0 | 1 | | 0.7 | | 4 | | 1.04 |
| Myliobatidae | *Aetobatus narinari* | Mobile invertivore | Y | - | 0.0 | - | 0.0 | - | 0.0 |  | 1 | 1.0 | 3 | 2.1 | 4 | | 1.4 | | 4 | | 0.69 |
| Myliobatidae | *Myliobatis* spp. | Mobile invertivore | Y | - | 0.0 | 2 | 4.2 | 2 | 1.4 |  | - | 0.0 | - | 0.0 | - | | 0.0 | | 2 | | 0.69 |
| Rhinopteridae | *Rhinoptera* spp. | Mobile invertivore | Y | 3 | 3.1 | - | 0.0 | 3 | 2.1 |  | 2 | 1.0 | - | 0.0 | 2 | | 0.7 | | 5 | | 1.39 |
| Albulidae | *Albula vulpes* | Mobile invertivore | Y | - | 0.0 | - | 0.0 | - | 0.0 |  | 1 | 1.0 | - | 0.0 | 1 | | 0.7 | | 1 | | 0.35 |
| Muraenidae | *Gymnothorax moringa* | Carnivore | Y | 4 | 4.2 | 10 | 18.8 | 14 | 9.0 |  | 1 | 1.0 | 5 | 10.4 | 6 | | 4.2 | | 20 | | 6.60 |
| Muraenidae | *Gymnothorax funebris* | Carnivore | Y | 4 | 4.2 | 3 | 6.3 | 7 | 4.9 |  | 1 | 1.0 | 3 | 6.3 | 4 | | 2.8 | | 11 | | 3.82 |
| Muraenidae | *Gymnothorax vicinus* | Carnivore | Y | - | 0.0 | 1 | 2.1 | 1 | 0.7 |  | - | 0.0 | - | 0.0 | - | | 0.0 | | 1 | | 0.35 |
| Synodontidae | *Synodus* spp. | Piscivore | N | 1 | 1.0 | 2 | 2.1 | 3 | 1.4 |  | - | 0.0 | 1 | 2.1 | 1 | | 0.7 | | 4 | | 1.04 |
| Synodontidae | *Synodus intermedius* | Piscivore | N | 1 | 1.0 | 2 | 4.2 | 3 | 2.1 |  | - | 0.0 | 2 | 2.1 | 2 | | 0.7 | | 5 | | 1.39 |
| Ogcocephalidae | *Ogcocephalus vespertilio* | Mobile invertivore | N | 2 | 2.1 | 1 | 2.1 | 3 | 2.1 |  | - | 0.0 | 1 | 2.1 | 1 | | 0.7 | | 4 | | 1.39 |
| Holocentridae | *Holocentrus adscensionis* | Mobile invertivore | N | 63 | 38.5 | 215 | 70.8 | 278 | 49.3 |  | 9 | 2.1 | 40 | 43.8 | 49 | | 16.0 | | 327 | | 32.64 |
| Fistulariidae | *Fistularia tabacaria* | Piscivore | Y | - | 0.0 | - | 0.0 | - | 0.0 |  | - | 0.0 | 2 | 4.2 | 2 | | 1.4 | | 2 | | 0.69 |
| Fistulariidae | *Fistularia petimba* | Piscivore | Y | - | 0.0 | - | 0.0 | - | 0.0 |  | - | 0.0 | 2 | 4.2 | 2 | | 1.4 | | 2 | | 0.69 |
| Dactylopteridae | *Dactylopterus volitans* | Mobile invertivore | Y | - | 0.0 | - | 0.0 | - | 0.0 |  | - | 0.0 | 9 | 10.4 | 9 | | 3.5 | | 9 | | 1.74 |
| Serranidae | *Serranus* spp. | Mobile invertivore | N | 1 | 1.0 | 3 | 6.3 | 4 | 2.8 |  | - | 0.0 | 1 | 2.1 | 1 | | 0.7 | | 5 | | 1.74 |
| Serranidae | *Serranus flaviventris* | Mobile invertivore | N | 8 | 6.3 | - | 0.0 | 8 | 4.2 |  | 2 | 2.1 | - | 0.0 | 2 | | 1.4 | | 10 | | 2.78 |
| Serranidae | *Serranus baldwini* | Mobile invertivore | N | 12 | 9.4 | 16 | 18.8 | 28 | 12.5 |  | - | 0.0 | 20 | 22.9 | 20 | | 7.6 | | 48 | | 10.07 |
| Serranidae | *Serranus atrobranchus* | Mobile invertivore | N | 2 | 2.1 | - | 0.0 | 2 | 1.4 |  | - | 0.0 | - | 0.0 | - | | 0.0 | | 2 | | 0.69 |
| Serranidae | *Diplectrum radiale* | Carnivore | N | 27 | 6.3 | 7 | 8.3 | 34 | 6.9 |  | - | 0.0 | 10 | 6.3 | 10 | | 2.1 | | 44 | | 4.51 |
| Epinephelidae | *Mycteroperca acutirostris* | Carnivore | Y | 35 | 31.3 | 8 | 12.5 | 43 | 25.0 |  | 49 | 35.4 | 20 | 31.3 | 69 | | 34.0 | | 112 | | 29.51 |
| Epinephelidae | *Mycteroperca marginatus* (EN_I_;VU_Br_) | Carnivore | Y | 133 | 61.5 | 44 | 52.1 | 177 | 58.3 |  | 9 | 8.3 | 15 | 18.8 | | 24 | | 11.8 | | 201 | 35.07 |
| Epinephelidae | *Mycteroperca bonaci* (NT_I_;VU_Br_) | Carnivore | Y | 3 | 3.1 | - | 0.0 | 3 | 2.1 |  | 1 | 1.0 | 2 | 2.1 | 3 | | 1.4 | | 6 | | 1.74 |
|  |  |  |  |  |  |  |  |  |  |  |  |  |  |  | Continue… | | | | | | |
| **Family** | **Species** | **Functional**  **group** | **Target** | **No-take** | | | | | |  | **Open** | | | | | | | | | **TOTAL N** | **TOTAL F%** |
|  |  |  |  | **Inshore** | | **Offshore** | | **Total N** | **Total F%** |  | **Inshore** | | **Offshore** | | | **Total N** | | **Total F%** | |  |  |
|  |  |  |  | **N** | **F%** | **N** | **F%** |  |  |  | **N** | **F%** | **N** | **F%** | |  |  |  |  |  |  |
| Epinephelidae | *Epinephelus morio* (NT_I_;VU_Br_) | Carnivore | Y | 7 | 7.3 | 1 | 2.1 | 8 | 5.6 |  | 3 | 3.1 | 1 | 2.1 | | 4 | | 2.8 | | 12 | 4.17 |
| Epinephelidae | *Cephalopholis* spp. | Carnivore | Y | - | 0.0 | 4 | 2.1 | 4 | 0.7 |  | - | 0.0 | - | 0.0 | | - | | 0.0 | | 4 | 0.35 |
| Epinephelidae | *Cephalopholis fulva* | Carnivore | Y | 1 | 1.0 | 1 | 2.1 | 2 | 1.4 |  | - | 0.0 | - | 0.0 | | - | | 0.0 | | 2 | 0.69 |
| Priacanthidae | *Heteropriacanthus cruentatus* | Mobile invertivore | Y | 14 | 4.2 | 1 | 2.1 | 15 | 3.5 |  | - | 0.0 | 38 | 8.3 | | 38 | | 2.8 | | 53 | 3.13 |
| Echeneidae | *Echeneis naucrates* | Planktivore | N | - | 0.0 | - | 0.0 | - | 0.0 |  | - | 0.0 | 1 | 2.1 | | 1 | | 0.7 | | 1 | 0.35 |
| Malacanthidae | *Malacanthus plumieri* | Carnivore | N | - | 0.0 | 16 | 20.8 | 16 | 6.9 |  | - | 0.0 | 1 | 2.1 | | 1 | | 0.7 | | 17 | 3.82 |
| Rachycentridae | *Rachycentron canadum* | Carnivore | Y | - | 0.0 | 1 | 2.1 | 1 | 0.7 |  | - | 0.0 | - | 0.0 | | - | | 0.0 | | 1 | 0.35 |
| Carangidae | *Caranx bartholomaei* | Carnivore | Y | 1 | 1.0 | 2 | 4.2 | 3 | 2.1 |  | 2 | 1.0 | 1 | 2.1 | | 3 | | 1.4 | | 6 | 1.74 |
| Carangidae | *Caranx crysos* | Carnivore | Y | 4 | 4.2 | 20 | 10.4 | 24 | 6.3 |  | 1 | 1.0 | 39 | 4.2 | | 40 | | 2.1 | | 64 | 4.17 |
| Carangidae | *Caranx hippos* | Carnivore | Y | 2 | 1.0 | 1 | 2.1 | 3 | 1.4 |  | - | 0.0 | - | 0.0 | | - | | 0.0 | | 3 | 0.69 |
| Carangidae | *Caranx latus* | Carnivore | Y | 114 | 20.8 | 46 | 27.1 | 160 | 22.9 |  | 96 | 18.8 | 40 | 31.3 | | 136 | | 22.9 | | 296 | 22.92 |
| Carangidae | *Pseudocaranx dentex* | Mobile invertivore | Y | 42 | 6.3 | 66 | 16.7 | 108 | 9.7 |  | 8 | 1.0 | 5 | 4.2 | | 13 | | 2.1 | | 121 | 5.90 |
| Carangidae | *Seriola* spp. | Carnivore | Y | 1 | 1.0 | 12 | 2.1 | 13 | 1.4 |  | - | 0.0 | - | 0.0 | | - | | 0.0 | | 13 | 0.69 |
| Carangidae | *Seriola dumerili* | Carnivore | Y | 3 | 1.0 | 3 | 6.3 | 6 | 2.8 |  | - | 0.0 | - | 0.0 | | - | | 0.0 | | 6 | 1.39 |
| Carangidae | *Seriola lalandi* | Carnivore | Y | - | 0.0 | 5 | 4.2 | 5 | 1.4 |  | - | 0.0 | - | 0.0 | | - | | 0.0 | | 5 | 0.69 |
| Carangidae | *Seriola rivoliana* | Carnivore | Y | 3 | 2.1 | 4 | 8.3 | 7 | 4.2 |  | - | 0.0 | - | 0.0 | | - | | 0.0 | | 7 | 2.08 |
| Carangidae | *Selene vomer* | Carnivore | Y | 2 | 1.0 | - | 0.0 | 2 | 0.7 |  | 3 | 2.1 | - | 0.0 | | 3 | | 1.4 | | 5 | 1.04 |
| Carangidae | *Decapterus* spp. | Planktivore | Y | 500 | 1.0 | 504 | 2.1 | 1004 | 1.4 |  | - | 0.0 | - | 0.0 | | - | | 0.0 | | 1004 | 0.69 |
| Carangidae | *Decapterus macarellus* | Planktivore | Y | 2 | 1.0 | - | 0.0 | 2 | 0.7 |  | - | 0.0 | 1 | 2.1 | | 1 | | 0.7 | | 3 | 0.69 |
| Carangidae | *Decapterus punctatus* | Planktivore | Y | - | 0.0 | 2304 | 4.2 | 2304 | 1.4 |  | - | 0.0 | - | 0.0 | | - | | 0.0 | | 2304 | 0.69 |
| Carangidae | *Chloroscombrus chrysurus* | Carnivore | Y | - | 0.0 | - | 0.0 | - | 0.0 |  | 2 | 1.0 | - | 0.0 | | 2 | | 0.7 | | 2 | 0.35 |
| Carangidae | *Trachinotus carolinus* | Carnivore | Y | 1 | 1.0 | - | 0.0 | 1 | 0.7 |  | - | 0.0 | - | 0.0 | | - | | 0.0 | | 1 | 0.35 |
| Carangidae | *Trachinotus goodei* | Carnivore | Y | 1 | 1.0 | - | 0.0 | 1 | 0.7 |  | - | 0.0 | - | 0.0 | | - | | 0.0 | | 1 | 0.35 |
| Carangidae | *Elagatis bipinnulata* | Planktivore | Y | - | 0.0 | - | 0.0 | - | 0.0 |  | - | 0.0 | 1 | 2.1 | | 1 | | 0.7 | | 1 | 0.35 |
| Lutjanidae | *Lutjanus* spp. | Carnivore | Y | 1 | 1.0 | - | 0.0 | 1 | 0.7 |  | - | 0.0 | 1 | 2.1 | | 1 | | 0.7 | | 2 | 0.69 |
| Lutjanidae | *Lutjanus analis* (NT_I_) | Carnivore | Y | 4 | 3.1 | 4 | 8.3 | 8 | 4.9 |  | 2 | 2.1 | - | 0.0 | | 2 | | 1.4 | | 10 | 3.13 |
| Lutjanidae | *Lutjanus jocu* | Carnivore | Y | 1 | 1.0 | - | 0.0 | 1 | 0.7 |  | - | 0.0 | - | 0.0 | | - | | 0.0 | | 1 | 0.35 |
| Lutjanidae | *Lutjanus synagris* | Carnivore | Y | 4 | 3.1 | - | 0.0 | 4 | 2.1 |  | - | 0.0 | - | 0.0 | | - | | 0.0 | | 4 | 1.04 |
| Lutjanidae | *Lutjanus chrysurus* | Carnivore | Y | 5 | 4.2 | 1 | 2.1 | 6 | 3.5 |  | 2 | 1.0 | 1 | 2.1 | | 3 | | 1.4 | | 9 | 2.43 |
| Lutjanidae | *Rhomboplites aurorubens* (VU_I_) | Carnivore | Y | - | 0.0 | 1807 | 22.9 | 1807 | 7.6 |  | 20 | 1.0 | 1 | 2.1 | | 21 | | 1.4 | | 1828 | 4.51 |
| Gerreidae | *Eugerres brasilianus* | Sessile invertivore | Y | - | 0.0 | - | 0.0 | - | 0.0 |  | 1 | 1.0 | - | 0.0 | | 1 | | 0.7 | | 1 | 0.35 |
|  |  |  |  |  |  |  |  |  |  |  |  |  |  |  | | Continue… | | | | | |
| **Family** | **Species** | **Functional**  **group** | **Target** | **No-take** | | | | | |  | **Open** | | | | | | | | | **TOTAL N** | **TOTAL F%** |
|  |  |  |  | **Inshore** | | **Offshore** | | **Total N** | **Total F%** |  | **Inshore** | | **Offshore** | | | **Total N** | | **Total F%** | |  |  |
|  |  |  |  | **N** | **F%** | **N** | **F%** |  |  |  | **N** | **F%** | **N** | **F%** | |  |  |  |  |  |  |
| Haemulidae | *Haemulon* spp. | Mobile invertivore | Y | 240 | 3.1 | 630 | 8.3 | 870 | 4.9 |  | 651 | 4.2 | 500 | 2.1 | | 1151 | | 3.5 | | 2021 | 4.17 |
| Haemulidae | *Haemulon parra* | Mobile invertivore | Y | 161 | 15.6 | 218 | 18.8 | 379 | 16.7 |  | 76 | 19.8 | 4 | 4.2 | | 80 | | 14.6 | | 459 | 15.63 |
| Haemulidae | *Haemulon plumieri* | Mobile invertivore | Y | 1 | 1.0 | 4 | 8.3 | 5 | 3.5 |  | - | 0.0 | 2 | 4.2 | | 2 | | 1.4 | | 7 | 2.43 |
| Haemulidae | *Haemulon aurolineatum* | Mobile invertivore | Y | 873 | 72.9 | 4336 | 89.6 | 5209 | 78.5 |  | 168 | 26.0 | 1231 | 97.9 | | 1399 | | 50.0 | | 6608 | 64.24 |
| Haemulidae | *Haemulon steindachneri* | Mobile invertivore | Y | 27 | 10.4 | 16 | 16.7 | 43 | 12.5 |  | 29 | 13.5 | 2 | 4.2 | | 31 | | 10.4 | | 74 | 11.46 |
| Haemulidae | *Anisotremus* spp. | Mobile invertivore | Y | - | 0.0 | 9 | 4.2 | 9 | 1.4 |  | 10 | 1.0 | - | 0.0 | | 10 | | 0.7 | | 19 | 1.04 |
| Haemulidae | *Anisotremus surinamensis* | Mobile invertivore | Y | 28 | 19.8 | 9 | 8.3 | 37 | 16.0 |  | 10 | 8.3 | 1 | 2.1 | | 11 | | 6.3 | | 48 | 11.11 |
| Haemulidae | *Anisotremus virginicus* | Mobile invertivore | Y | 96 | 50.0 | 121 | 58.3 | 217 | 52.8 |  | 67 | 21.9 | 17 | 31.3 | | 84 | | 25.0 | | 301 | 38.89 |
| Sparidae | *Calamus* spp. | Omnivore | Y | - | 0.0 | 1 | 2.1 | 1 | 0.7 |  | - | 0.0 | - | 0.0 | | - | | 0.0 | | 1 | 0.35 |
| Sparidae | *Calamus pennatula* | Omnivore | Y | 1 | 1.0 | 9 | 8.3 | 10 | 3.5 |  | 3 | 1.0 | 1 | 2.1 | | 4 | | 1.4 | | 14 | 2.43 |
| Sparidae | *Calamus bajonado* | Omnivore | Y | - | 0.0 | 1 | 2.1 | 1 | 0.7 |  | - | 0.0 | - | 0.0 | | - | | 0.0 | | 1 | 0.35 |
| Sparidae | *Calamus penna* | Omnivore | Y | - | 0.0 | 2 | 4.2 | 2 | 1.4 |  | - | 0.0 | - | 0.0 | | - | | 0.0 | | 2 | 0.69 |
| Sparidae | *Diplodus argenteus* | Omnivore | Y | 83 | 28.1 | 44 | 43.8 | 127 | 33.3 |  | 9 | 8.3 | 61 | 39.6 | | 70 | | 18.8 | | 197 | 26.04 |
| Sciaenidae | *Odontoscion dentex* | Carnivore | Y | 14 | 11.5 | 13 | 14.6 | 27 | 12.5 |  | 17 | 4.2 | 2 | 4.2 | | 19 | | 4.2 | | 46 | 8.33 |
| Sciaenidae | *Pareques acuminatus* | Mobile invertivore | N | 19 | 10.4 | 2 | 4.2 | 21 | 8.3 |  | 10 | 7.3 | 4 | 6.3 | | 14 | | 6.9 | | 35 | 7.64 |
| Mullidae | *Pseudupeneus maculatus* | Mobile invertivore | Y | 17 | 12.5 | 5 | 8.3 | 22 | 11.1 |  | 8 | 7.3 | 21 | 33.3 | | 29 | | 16.0 | | 51 | 13.54 |
| Mullidae | *Mullus argentinae* | Carnivore | Y | - | 0.0 | - | 0.0 | - | 0.0 |  | - | 0.0 | 1 | 2.1 | | 1 | | 0.7 | | 1 | 0.35 |
| Pempheridae | *Pempheris schomburgkii* | Planktivore | N | 61 | 2.1 | 2 | 2.1 | 63 | 2.1 |  | 20 | 1.0 | - | 0.0 | | 20 | | 0.7 | | 83 | 1.39 |
| Kyphosidae | *Kyphosus* spp. | Roving herbivore | Y | 60 | 21.9 | 96 | 62.5 | 156 | 35.4 |  | 12 | 1.0 | 36 | 31.3 | | 48 | | 11.1 | | 204 | 23.26 |
| Kyphosidae | *Kyphosus sectatrix* | Roving herbivore | Y | - | 0.0 | 1 | 2.1 | 1 | 0.7 |  | - | 0.0 | - | 0.0 | | - | | 0.0 | | 1 | 0.35 |
| Kyphosidae | *Kyphosus incisor* | Roving herbivore | Y | - | 0.0 | 4 | 4.2 | 4 | 1.4 |  | - | 0.0 | 3 | 2.1 | | 3 | | 0.7 | | 7 | 1.04 |
| Chaetodontidae | *Chaetodon sedentarius* | Sessile invertivore | N | 4 | 1.0 | - | 0.0 | 4 | 0.7 |  | - | 0.0 | - | 0.0 | | - | | 0.0 | | 4 | 0.35 |
| Chaetodontidae | *Chaetodon striatus* | Sessile invertivore | N | 47 | 26.0 | 24 | 29.2 | 71 | 27.1 |  | 41 | 18.8 | 50 | 58.3 | | 91 | | 31.9 | | 162 | 29.51 |
| Pomacanthidae | *Pomacanthus paru* | Omnivore | N | 59 | 34.4 | 94 | 75.0 | 153 | 47.9 |  | 10 | 6.3 | 31 | 43.8 | | 41 | | 18.8 | | 194 | 33.33 |
| Pomacanthidae | *Holacanthus tricolor* | Sessile invertivore | N | 2 | 2.1 | - | 0.0 | 2 | 1.4 |  | - | 0.0 | 2 | 4.2 | | 2 | | 1.4 | | 4 | 1.39 |
| Pomacentridae | *Abudefduf saxatilis* | Omnivore | N | 604 | 42.7 | 424 | 45.8 | 1028 | 43.8 |  | 415 | 47.9 | 939 | 70.8 | | 1354 | | 55.6 | | 2382 | 49.65 |
| Pomacentridae | *Chromis multilineata* | Planktivore | N | 50 | 21.9 | 96 | 54.2 | 146 | 32.6 |  | 18 | 2.1 | 40 | 33.3 | | 58 | | 12.5 | | 204 | 22.57 |
| Pomacentridae | *Chromis jubauna** | Planktivore | N | - | 0.0 | 11 | 12.5 | 11 | 4.2 |  | - | 0.0 | 7 | 6.3 | | 7 | | 2.1 | | 18 | 3.13 |
| Pomacentridae | *Chromis enchrysura* | Planktivore | N | - | 0.0 | 3 | 2.1 | 3 | 0.7 |  | - | 0.0 | - | 0.0 | | - | | 0.0 | | 3 | 0.35 |
| Pomacentridae | *Stegastes fuscus** | Territorial herbivore | N | 265 | 53.1 | 226 | 60.4 | 491 | 55.6 |  | 335 | 45.8 | 285 | 66.7 | | 620 | | 52.8 | | 1111 | 54.17 |
| Pomacentridae | *Stegastes pictus** | Territorial herbivore | N | 3 | 2.1 | 9 | 8.3 | 12 | 4.2 |  | - | 0.0 | - | 0.0 | | - | | 0.0 | | 12 | 2.08 |
|  |  |  |  |  |  |  |  |  |  |  |  |  |  |  | | Continue… | | | | | |
| **Family** | **Species** | **Functional**  **group** | **Target** | **No-take** | | | | | |  | **Open** | | | | | | | | | **TOTAL N** | **TOTAL F%** |
|  |  |  |  | **Inshore** | | **Offshore** | | **Total N** | **Total F%** |  | **Inshore** | | **Offshore** | | | **Total N** | | **Total F%** | |  |  |
|  |  |  |  | **N** | **F%** | **N** | **F%** |  |  |  | **N** | **F%** | **N** | **F%** | |  |  |  |  |  |  |
| Pomacentridae | *Stegastes variabilis** | Territorial herbivore | N | - | 0.0 | 3 | 4.2 | 3 | 1.4 |  | 1 | 1.0 | - | 0.0 | | 1 | | 0.7 | | 4 | 1.04 |
| Sphyraenidae | *Sphyraena guachancho* | Carnivore | Y | 1 | 1.0 | - | 0.0 | 1 | 0.7 |  | - | 0.0 | - | 0.0 | | - | | 0.0 | | 1 | 0.35 |
| Labridae | *Bodianus pulchellus* | Mobile invertivore | Y | 6 | 6.3 | 37 | 52.1 | 43 | 21.5 |  | 2 | 1.0 | 39 | 52.1 | | 41 | | 18.1 | | 84 | 19.79 |
| Labridae | *Bodianus rufus* | Mobile invertivore | Y | 21 | 16.7 | 24 | 35.4 | 45 | 22.9 |  | 2 | 1.0 | 22 | 37.5 | | 24 | | 13.2 | | 69 | 18.06 |
| Labridae | *Halichoeres* spp. | Mobile invertivore | Y | 1 | 1.0 | - | 0.0 | 1 | 0.7 |  | - | 0.0 | - | 0.0 | | - | | 0.0 | | 1 | 0.35 |
| Labridae | *Halichoeres brasiliensis** | Mobile invertivore | Y | 2 | 2.1 | 10 | 20.8 | 12 | 8.3 |  | - | 0.0 | 10 | 20.8 | | 10 | | 6.9 | | 22 | 7.64 |
| Labridae | *Halichoeres poeyi* | Mobile invertivore | N | 20 | 14.6 | 22 | 27.1 | 42 | 18.8 |  | 24 | 15.6 | 121 | 68.8 | | 145 | | 33.3 | | 187 | 26.04 |
| Labridae | *Halichoeres sazimai** | Mobile invertivore | N | - | 0.0 | - | 0.0 | - | 0.0 |  | - | 0.0 | 1 | 2.1 | | 1 | | 0.7 | | 1 | 0.35 |
| Labridae | *Halichoeres dimidiatus** | Mobile invertivore | N | - | 0.0 | 3 | 4.2 | 3 | 1.4 |  | - | 0.0 | - | 0.0 | | - | | 0.0 | | 3 | 0.69 |
| Labridae | *Halichoeres bivittatus* | Mobile invertivore | N | - | 0.0 | 8 | 4.2 | 8 | 1.4 |  | - | 0.0 | - | 0.0 | | - | | 0.0 | | 8 | 0.69 |
| Labridae | *Halichoeres penrosei** | Mobile invertivore | N | - | 0.0 | - | 0.0 | - | 0.0 |  | - | 0.0 | 1 | 2.1 | | 1 | | 0.7 | | 1 | 0.35 |
| Labridae | *Clepticus brasiliensis** | Planktivore | N | 1 | 1.0 | 1 | 2.1 | 2 | 1.4 |  | - | 0.0 | - | 0.0 | | - | | 0.0 | | 2 | 0.69 |
| Scaridae | *Sparisoma* spp. | Roving herbivore | Y | 1 | 1.0 | 2 | 2.1 | 3 | 1.4 |  | - | 0.0 | - | 0.0 | | - | | 0.0 | | 3 | 0.69 |
| Scaridae | *Sparisoma amplum** | Roving herbivore | Y | 5 | 4.2 | - | 0.0 | 5 | 2.8 |  | - | 0.0 | - | 0.0 | | - | | 0.0 | | 5 | 1.39 |
| Scaridae | *Sparisoma axillare** (VU_Br_) | Roving herbivore | Y | 14 | 10.4 | 23 | 25.0 | 37 | 15.3 |  | 1 | 1.0 | 53 | 33.3 | | 54 | | 11.8 | | 91 | 13.54 |
| Scaridae | *Sparisoma frondosum** (VU_Br_) | Roving herbivore | Y | 14 | 8.3 | 11 | 12.5 | 25 | 9.7 |  | 12 | 1.0 | 22 | 20.8 | | 34 | | 7.6 | | 59 | 8.68 |
| Scaridae | *Sparisoma tuiupiranga** | Roving herbivore | Y | 1 | 1.0 | 10 | 14.6 | 11 | 5.6 |  | 1 | 1.0 | 20 | 10.4 | | 21 | | 4.2 | | 32 | 4.86 |
| Scaridae | *Sparisoma radians* | Roving herbivore | Y | - | 0.0 | - | 0.0 | - | 0.0 |  | - | 0.0 | 4 | 4.2 | | 4 | | 1.4 | | 4 | 0.69 |
| Scaridae | *Scarus trispinosus** (EN_I_;EN_BR_) | Roving herbivore | Y | - | 0.0 | - | 0.0 | - | 0.0 |  | - | 0.0 | 1 | 2.1 | | 1 | | 0.7 | | 1 | 0.35 |
| Scaridae | *Scarus zelindae** (VU_Br_) | Roving herbivore | Y | 1 | 1.0 | - | 0.0 | 1 | 0.7 |  | - | 0.0 | 5 | 8.3 | | 5 | | 2.8 | | 6 | 1.74 |
| Scaridae | *Cryptotomus roseus* | Roving herbivore | N | - | 0.0 | 1 | 2.1 | 1 | 0.7 |  | - | 0.0 | 26 | 6.3 | | 26 | | 2.1 | | 27 | 1.39 |
| Labrisomidae | *Labrisomus nuchipinnis* | Mobile invertivore | N | - | 0.0 | - | 0.0 | - | 0.0 |  | 8 | 8.3 | - | 0.0 | | 8 | | 5.6 | | 8 | 2.78 |
| Labrisomidae | *Malacoctenus delalandii* | Mobile invertivore | N | 3 | 3.1 | - | 0.0 | 3 | 2.1 |  | 7 | 7.3 | 1 | 2.1 | | 8 | | 5.6 | | 11 | 3.82 |
| Chaenopsidae | *Emblemariopsis signifer** | Mobile invertivore | N | 46 | 16.7 | 10 | 12.5 | 56 | 15.3 |  | 1 | 1.0 | 7 | 8.3 | | 8 | | 3.5 | | 64 | 9.38 |
| Blenniidae | *Parablennius* spp. | Omnivore | N | 39 | 13.5 | 18 | 18.8 | 57 | 15.3 |  | 8 | 6.3 | 3 | 4.2 | | 11 | | 5.6 | | 68 | 10.42 |
| Blenniidae | *Parablennius marmoreus* | Omnivore | N | - | 0.0 | 3 | 6.3 | 3 | 2.1 |  | - | 0.0 | - | 0.0 | | - | | 0.0 | | 3 | 1.04 |
| Blenniidae | *Parablennius pilicornis* | Omnivore | N | - | 0.0 | 1 | 2.1 | 1 | 0.7 |  | - | 0.0 | - | 0.0 | | - | | 0.0 | | 1 | 0.35 |
| Blenniidae | *Scartella cristata* | Territorial herbivore | N | 2 | 2.1 | - | 0.0 | 2 | 1.4 |  | - | 0.0 | - | 0.0 | | - | | 0.0 | | 2 | 0.69 |
| Gobiidae | *Coryphopterus* spp. | Mobile invertivore | N | 289 | 32.3 | 227 | 37.5 | 516 | 34.0 |  | 3 | 2.1 | 27 | 20.8 | | 30 | | 8.3 | | 546 | 21.18 |
| Gobiidae | *Coryphopterus glaucofraenum* | Mobile invertivore | N | - | 0.0 | 6 | 4.2 | 6 | 1.4 |  | - | 0.0 | 1 | 2.1 | | 1 | | 0.7 | | 7 | 1.04 |
| Gobiidae | *Coryphopterus thrix* (VU_i_) | Mobile invertivore | N | 2 | 1.0 | - | 0.0 | 2 | 0.7 |  | - | 0.0 | - | 0.0 | | - | | 0.0 | | 2 | 0.35 |
|  |  |  |  |  |  |  |  |  |  |  |  |  |  |  | | Continue… | | | | | |
| **Family** | **Species** | **Functional**  **group** | **Target** | **No-take** | | | | | |  | **Open** | | | | | | | | | **TOTAL N** | **TOTAL F%** |
|  |  |  |  | **Inshore** | | **Offshore** | | **Total N** | **Total F%** |  | **Inshore** | | **Offshore** | | | **Total N** | | **Total F%** | |  |  |
|  |  |  |  | **N** | **F%** | **N** | **F%** |  |  |  | **N** | **F%** | **N** | **F%** | |  |  |  |  |  |  |
| Gobiidae | *Elacatinus figaro** (VU_Br_) | Mobile invertivore | N | 7 | 5.2 | 19 | 8.3 | 26 | 6.3 |  | 2 | 1.0 | - | 0.0 | | 2 | | 0.7 | | 28 | 3.47 |
| Ephippidae | *Chaetodipterus faber* | Omnivore | Y | 57 | 6.3 | 91 | 14.6 | 148 | 9.0 |  | 6 | 2.1 | 4 | 4.2 | | 10 | | 2.8 | | 158 | 5.90 |
| Acanthuridae | *Acanthurus* spp. | Roving herbivore | N | 3 | 3.1 | 25 | 8.3 | 28 | 4.9 |  | 2 | 1.0 | 12 | 14.6 | | 14 | | 5.6 | | 42 | 5.21 |
| Acanthuridae | *Acanthurus bahianus** | Roving herbivore | N | 4 | 4.2 | 33 | 22.9 | 37 | 10.4 |  | - | 0.0 | 45 | 20.8 | | 45 | | 6.9 | | 82 | 8.68 |
| Acanthuridae | *Acanthurus chirurgus* | Roving herbivore | N | 44 | 14.6 | 26 | 12.5 | 70 | 13.9 |  | 4 | 1.0 | 40 | 22.9 | | 44 | | 8.3 | | 114 | 11.11 |
| Mugilidae | *Mugil* spp. | Omnivore | Y | 239 | 7.3 | - | 0.0 | 239 | 4.9 |  | 34 | 8.3 | 264 | 14.6 | | 298 | | 10.4 | | 537 | 7.64 |
| Mugilidae | *Mugil liza* | Omnivore | Y | 7 | 2.1 | - | 0.0 | 7 | 1.4 |  | - | 0.0 | - | 0.0 | | - | | 0.0 | | 7 | 0.69 |
| Mugilidae | *Mugil curema* | Omnivore | Y | 104 | 2.1 | - | 0.0 | 104 | 1.4 |  | 25 | 3.1 | 5 | 2.1 | | 30 | | 2.8 | | 134 | 2.08 |
| Bothidae | *Bothus ocellatus* | Carnivore | N | 1 | 1.0 | 3 | 2.1 | 4 | 1.4 |  | - | 0.0 | 5 | 6.3 | | 5 | | 2.1 | | 9 | 1.74 |
| Bothidae | *Bothus* spp. | Carnivore | N | - | 0.0 | - | 0.0 | - | 0.0 |  | - | 0.0 | 4 | 4.2 | | 4 | | 1.4 | | 4 | 0.69 |
| Balistidae | *Balistes vetula* (NT_I_) | Omnivore | Y | 1 | 1.0 | - | 0.0 | 1 | 0.7 |  | 2 | 1.0 | 7 | 12.5 | | 9 | | 4.9 | | 10 | 2.78 |
| Balistidae | *Balistes capriscus* (VU_I_) | Omnivore | Y | - | 0.0 | - | 0.0 | - | 0.0 |  | 2 | 1.0 | 1 | 2.1 | | 3 | | 1.4 | | 3 | 0.69 |
| Monacanthidae | *Cantherhines pullus* | Omnivore | N | 1 | 1.0 | 9 | 12.5 | 10 | 4.9 |  | 8 | 1.0 | 9 | 16.7 | | 17 | | 6.3 | | 27 | 5.56 |
| Monacanthidae | *Cantherhines macrocerus* | Omnivore | N | - | 0.0 | 9 | 14.6 | 9 | 4.9 |  | - | 0.0 | 3 | 4.2 | | 3 | | 1.4 | | 12 | 3.13 |
| Monacanthidae | *Aluterus monoceros* | Omnivore | N | 7 | 2.1 | - | 0.0 | 7 | 1.4 |  | - | 0.0 | 20 | 2.1 | | 20 | | 0.7 | | 27 | 1.04 |
| Monacanthidae | *Aluterus scriptus* | Omnivore | N | - | 0.0 | 3 | 4.2 | 3 | 1.4 |  | - | 0.0 | - | 0.0 | | - | | 0.0 | | 3 | 0.69 |
| Monacanthidae | *Stephanolepis hispidus* | Mobile invertivore | N | - | 0.0 | - | 0.0 | - | 0.0 |  | - | 0.0 | 3 | 6.3 | | 3 | | 2.1 | | 3 | 1.04 |
| Ostraciidae | *Acanthostracion polygonius* | Omnivore | N | - | 0.0 | 1 | 2.1 | 1 | 0.7 |  | - | 0.0 | - | 0.0 | | - | | 0.0 | | 1 | 0.35 |
| Tetraodontidae | *Canthigaster figueiredoi** | Omnivore | N | 3 | 3.1 | 7 | 10.4 | 10 | 5.6 |  | - | 0.0 | 5 | 8.3 | | 5 | | 2.8 | | 15 | 4.17 |
| Tetraodontidae | *Sphoeroides spengleri* | Mobile invertivore | N | 2 | 2.1 | 1 | 2.1 | 3 | 2.1 |  | 3 | 3.1 | 9 | 14.6 | | 12 | | 6.9 | | 15 | 4.51 |
| Tetraodontidae | *Sphoeroides greeleyi* | Mobile invertivore | N | - | 0.0 | - | 0.0 | - | 0.0 |  | 11 | 10.4 | - | 0.0 | | 11 | | 6.9 | | 11 | 3.47 |
